# Analysis of risk factors for Meige syndrome and construction and validation of a clinical prediction nomogram model

**DOI:** 10.3389/fneur.2026.1755240

**Published:** 2026-02-03

**Authors:** Gang Liu, Qiangying Guo, Jie Xiang, Zhen Xu, Suying Chen, Lili Shang, Jiao Du, Huiying Wang, Xianzhong Liu, Yongjun Wu

**Affiliations:** 1 Meige Syndrome Center, The Third People’s Hospital of Henan Province, Zhengzhou, China; 2 School of Public Health, Zhengzhou University, Zhengzhou, China

**Keywords:** blepharospasm, Meige syndrome, nomogram, prediction mode, risk factors

## Abstract

**Background:**

Meige syndrome (MS) is a craniocervical dystonia characterized by blepharospasm and oromandibular dystonia. Its etiology remains unclear, and clinical diagnosis is often delayed. Currently, there is a lack of effective risk prediction tools, making early intervention challenging.

**Objective:**

To systematically analyze the risk factors for MS and develop and validate a clinical prediction nomogram model based on clinical indicators to facilitate early risk assessment.

**Methods:**

A retrospective case–control study was conducted, enrolling 450 confirmed MS patients and 450 controls from the Third People’s Hospital of Henan Province between January 2021 and December 2023. Univariate and multivariate logistic regression analyses were performed to identify independent risk factors, and a nomogram prediction model was constructed based on regression coefficients. The model’s discriminative ability, calibration, and clinical utility were evaluated using receiver operating characteristic curves, calibration curves, and decision curve analysis (DCA).

**Results:**

Multivariate analysis revealed that a history of thyroid disease (OR = 12.797), psychiatric disorders (OR = 6.892), and head/face surgery (OR = 3.466) were independent risk factors for MS, while female sex (OR = 1.87) and cerebrovascular disease (OR = 1.999) were moderate-risk factors. Notably, smoking (OR = 0.411), alcohol consumption (OR = 0.396), and diabetes (OR = 0.534) showed protective associations. The constructed nomogram model demonstrated strong predictive performance in both the training and validation sets (AUC = 0.789 and 0.800, respectively). Calibration curves indicated high consistency between predicted and observed probabilities, and DCA confirmed its clinical applicability.

**Conclusion:**

We developed and validated a clinical prediction nomogram for MS incorporating eight independent predictors: history of thyroid disorders, psychiatric disorders, head/face surgery, female sex, cerebrovascular disease, as well as protective factors including smoking, alcohol consumption, and diabetes. The model provides a quantifiable tool for early risk stratification and targeted intervention in clinical practice. However, further optimization and validation through multicenter prospective studies are warranted.

## 1 Introduction

Meige syndrome (MS) is a segmental craniocervical dystonia characterized primarily by blepharospasm and oromandibular dystonia (1). Clinically, it presents with progressive bilateral blepharospasm, which may lead to functional blindness, accompanied by oromandibular involuntary movements such as teeth grinding and lip pursing. As the disease progresses, patients often develop masticatory and swallowing difficulties, abnormal neck postures, and psychiatric symptoms such as anxiety and depression, collectively leading to significant quality-of-life impairment. Epidemiologically, MS exhibits marked geographical variation, with prevalence rates ranging from 43.97 per 100,000 in Sweden to 2.70 per 100,000 in China, and a consistently higher risk observed in women (2). Importantly, due to the nonspecific nature of its early manifestations, clinical diagnosis is frequently delayed, with up to 62.5% of patients identified beyond the optimal therapeutic window (3). This diagnostic challenge underscores a critical gap in the early recognition of MS, pointing to the need for more objective and integrative assessment approaches (4, 5).

Despite being recognized for over a century, the etiological mechanisms of MS remain incompletely understood. Current evidence suggests that genetic predisposition, environmental triggers, and neurochemical imbalances may all contribute to its pathogenesis, yet their specific interactions and relative importance remain poorly defined (6). Existing studies on its risk factors are predominantly limited to small-scale, single-center analyses, which often yield inconsistent results and offer little insight into potential protective factors. This fragmented understanding, combined with a lack of integration across multidimensional clinical and biological indicators, has resulted in a clear clinical gap: there is currently no validated predictive tool available for identification and risk stratification.

To address this unmet need, a multifactorial approach integrating diverse clinical parameters is essential for advancing toward early identification and individualized prevention. Therefore, the primary objective of this study was to develop and validate a clinical prediction nomogram for assessing MS risk based on systematically identified risk factors. This tool aims to provide clinicians with a practical, visually intuitive means to estimate individualized risk, thereby facilitating timely diagnosis and informed clinical decision-making.

## 2 Subjects and methods

### 2.1 Study subjects

This study employed a retrospective case–control design with a cross-sectional analytical approach. A total of 450 patients diagnosed with MS at the Meige Syndrome Center of the Third People’s Hospital of Henan Province between January 2021 and December 2023 were included as the case group. Meanwhile, 450 non-MS patients who were hospitalized in the same department during the same period were selected by simple random sampling as the control group.

*Inclusion criteria for MS group*: (1) Diagnosis by experienced specialists, meeting the following diagnostic criteria: Blepharospasm accompanied by oromandibular dystonia, presence of sensory tricks (geste antagoniste), and Symptoms disappearing during sleep (7); (2) Medical records were obtained through structured interviews by trained physicians, ensuring data reliability; (3) Complete electronic medical records were available. *Exclusion criteria for MS group*: (1) Patients with a record of two hospital admissions during the period from January 2021 to December 2023; (2) Unreliable medical records or obvious documentation errors.

*Inclusion criteria for control group*: (1) Randomly selected non-MS inpatients from the same department during the same period, aged ≥40 years; (2) No history of dystonia; (3) Complete electronic medical records. *Exclusion criteria for control group*: (1) Psychiatric disorders; (2) Abnormal findings on cranial MRI.

The mean disease duration for MS patients was 4.2 ± 3.1 years, defined as the time interval from self-reported symptom onset to the date of assessment during this admission. Information for all study variables was assessed based on their status prior to this admission.

The study was approved by the Ethics Committee of Third People’s Hospital of Henan Province (Approval No. 2025SZSYLCYJ0807), and all participants provided informed consent.

### 2.2 Study variables

A structured interview protocol was implemented by trained physicians to systematically collect clinical and demographic data, with all information recorded in the electronic medical record system.

#### 2.2.1 Dependent variable

The primary outcome was a diagnosis of MS, as clinically confirmed by movement disorder specialists according to the diagnostic criteria detailed in Section 2.1.

#### 2.2.2 Independent variables

Based on existing literature suggesting potential links to dystonia or cranial neuropathy, the following candidate predictors were selected and operationalized as follows:

*Demographic factors*: Age (continuous, years), Sex (male/female), Education Level (highest academic degree attained).

##### 2.2.2.1 Lifestyle and exposure history

*Smoking*: Defined per WHO criteria as cumulative consumption of ≥100 cigarettes over a period of ≥6 months (8).

*Alcohol consumption*: Classified as significant use if average daily ethanol intake was ≥40 g for men or ≥20 g for women, sustained for ≥1 year (8).

##### 2.2.2.2 Medical history

*Systemic conditions*: Documented history of hypertension, diabetes mellitus, coronary heart disease, thyroid disorders, hyperlipidemia, and cerebrovascular disease.

##### 2.2.2.3 Local cranial conditions

*Head/Face surgery*: History of any surgical procedure involving the cranial or facial region, excluding isolated ocular surgeries.

*Head/Face disorders*: Included sinusitis (ethmoid, frontal, maxillary), Ménière’s disease, rhinitis, and otitis media with effusion.

*Head/Face trauma*: Any documented history of trauma to the head or facial area.

##### 2.2.2.4 Psychiatric factors

*Psychiatric history*: Physician-documented diagnosis of anxiety disorders, depression, schizophrenia, or obsessive-compulsive disorder.

*Current mental status*: Evaluated at admission by nursing staff using a standardized three-tier classification (poor, suboptimal, good) (9).

*Medication history*: Documented use of antiparkinsonian medications.

*Family history*: Reported history of dystonia or related movement disorders in first-degree relatives.

### 2.3 Statistical methods

All analyses were performed using SPSS 25.0 and R 4.4.1 software. Data presentation and analytical approaches were selected based on variable characteristics: Categorical data (including gender, education level, mental status, smoking/drinking status, family history, and 12 clinical histories: hypertension, diabetes, coronary heart disease, thyroid disorders, hyperlipidemia, cerebrovascular disease, head/face surgery, head/face disorders, trauma, psychiatric conditions, and antiparkinsonian medication use) were presented as *n* (%), and intergroup comparisons employed the χ^2^ test. Continuous variables (age) were reported as means ± standard deviations and evaluated using two-tailed Student’s *t*-tests. To assess MS risk factors, we conducted both univariate and multivariate logistic regression analyses. Prior to multivariate analysis, multicollinearity among candidate predictors was assessed using the variance inflation factor (VIF), with a VIF value > 10 indicating severe collinearity requiring model adjustment. The cohort was randomly split into training (70%) and validation (30%) cohorts for model development and evaluation. Using R’s rms package, a nomogram was constructed by incorporating predictors that remained statistically significant in the multivariate model. The model’s discriminative ability was evaluated using the area under the receiver operating characteristic curve (AUC) in both training and validation sets. Model fit was primarily assessed through calibration performance, which reflects the agreement between predicted probabilities and observed outcomes. Calibration was visually examined using calibration plots and further internally validated via the bootstrap method with 1,000 resamples to correct for optimism and generate a bias-corrected calibration curve. Clinical utility was analyzed via decision curve analysis to evaluate the net benefit across a range of probability thresholds. A *p* value of < 0.05 indicates statistical significance.

## 3 Results

### 3.1 Univariate analysis of clinical characteristics

The comparative analysis revealed no statistically significant differences between the MS and control groups regarding age, family history, education level, hypertension, coronary heart disease, hyperlipidemia, history of head/face disorders, or trauma history. However, several notable differences emerged: the MS group demonstrated markedly higher proportions of Female predominance, Thyroid disorders, Cerebrovascular disease, Psychiatric disorders, History of head/face surgery, and Use of antiparkinsonian medications. Conversely, the MS group showed markedly lower rates of Smoking history, Alcohol consumption, and Diabetes mellitus (Table 1).

**Table 1:** Comparison of clinical characteristics [*n* (%), ( x− ± s)].

	MS group (*n* = 450)	Control group (*n* = 450)	*t*/*χ2*	*p*
Age (years)	63.1 ± 7.8	62.3 ± 9.9	1.346	0.178
Sex, *n* (%)				
Female	311 (69.1)	253 (56.2)	15.976	<0.001
Male	139 (30.9)	197 (43.8)		
Smoking history, *n* (%)	49 (10.9)	101 (22.4)	21.632	<0.001
Alcohol consumption, *n* (%)	51 (11.3)	103 (22.9)	21.183	<0.001
Family history, *n* (%)	3 (0.7)	1 (0.2)	1.004	0.316
Education Level, *n* (%)				
Junior high or below	281 (62.5)	306 (68.0)	−1.912	0.056
High school/vocational	100 (22.2)	93 (20.7)		
College or above	69 (15.3)	51 (11.3)		
Underlying disease, *n* (%)				
Hypertension	167 (37.1)	177 (39.3)	0.471	0.493
Diabetes mellitus	75 (16.7)	121 (26.9)	13.802	<0.001
Coronary heart disease	51 (11.3)	65 (14.4)	1.940	0.164
Thyroid disorders	177 (39.3)	22 (4.9)	155.001	<0.001
Hyperlipidemia	51 (11.3)	52 (11.6)	0.011	0.917
Cerebrovascular disease	81 (18.0)	52 (11.6)	7.420	0.006
Medical history, *n* (%)				
Head/face surgery	46 (10.2)	14 (3.1)	18.131	<0.001
Head/face disorders	8 (1.8)	14 (3.1)	1.677	0.195
Trauma history	27 (6.0)	33 (7.3)	0.643	0.423
Psychiatric disorders	23 (5.1)	5 (1.1)	11.943	<0.001
Antiparkinsonian drugs, *n* (%)	11 (2.4)	1 (0.2)	8.446	0.004
Mental status, *n* (%)				
Good	415 (92.2)	425 (94.4)	−1.280	0.201
Suboptimal	20 (4.4)	9 (2.0)		
Poor	15 (3.4)	16 (3.6)		

### 3.2 Risk factors and their association for MS

The following variables exhibited significant differences in univariate analysis and were assigned values: Female sex, Smoking history, Alcohol consumption history, Diabetes mellitus history, Thyroid disorders, Cerebrovascular disease history, History of head/face surgery, Psychiatric disorders history, Use of antiparkinsonian medications (Table 2).

**Table 2:** Risk factors and their variable assignments for complication.

Factor	Variable	Assignment
Sex	X1	Male, 0; Female, 1
Smoking history	X2	No, 0; Yes, 1
Alcohol consumption	X3	No, 0; Yes, 1
Diabetes mellitus	X4	No, 0; Yes, 1
Thyroid disorders	X5	No, 0; Yes, 1
Cerebrovascular disease	X6	No, 0; Yes, 1
Head/face surgery	X7	No, 0; Yes, 1
Psychiatric disorders	X8	No, 0; Yes, 1
Antiparkinsonian drugs	X9	No, 0; Yes, 1

### 3.3 Multivariate risk analysis of MS

Assessment of multicollinearity among the predictors selected for the multivariate model showed all VIF values were below 5, indicating no severe multicollinearity that would compromise the stability of the regression estimates.

Multivariate logistic regression analysis demonstrated that sex, smoking history, alcohol consumption, diabetes history, thyroid disorders, cerebrovascular disease, head/face surgery history, and psychiatric history were markedly associated with MS. Specifically, thyroid disorders, psychiatric history, and head/face surgery history emerged as independent risk factors, while smoking, alcohol consumption, and diabetes history showed protective associations. Additionally, female sex and cerebrovascular disease history were identified as moderate risk factors, whereas no statistically significant association was found between antiparkinsonian medication use and disease occurrence (Table 3).

**Table 3:** Multivariate risk analysis for MS.

	OR	95% CI	*P*
Sex	1.870	1.355–2.581	<0.001
Smoking history	0.411	0.264–0.639	<0.001
Alcohol consumption	0.396	0.257–0.609	<0.001
Diabetes mellitus	0.534	0.367–0.776	0.001
Thyroid disorders	12.797	7.856–20.845	<0.001
Cerebrovascular disease	1.999	1.294–3.088	0.002
Head/face surgery	3.466	1.774–6.774	<0.001
Psychiatric disorders	6.892	2.368–20.054	<0.001
Antiparkinsonian drugs	6.751	0.690–66.089	0.101

### 3.4 Development and validation of the MS prediction model

The original dataset was randomly divided into training and validation cohorts at a 7:3 ratio for nomogram construction and internal validation, with baseline characteristics of both cohorts presented in Table 4.

**Table 4:** Comparison of clinical characteristics between training and validation cohorts [*n* (%), ( x− ± s)].

	Training cohort (*n* = 675)	Validation cohort (*n* = 225)	*χ* ^2^	*p*
MS, *n* (%)				
No	341 (50.5)	109 (48.4)	0.290	0.589
Yes	334 (49.5)	116 (51.6)		
Sex, *n* (%)				
Female	417 (61.8)	147 (65.3)	0.912	0.340
Male	258 (38.2)	78 (34.7)		
Smoking history, *n* (%)	104 (15.4)	46 (20.9)	3.083	0.079
Alcohol consumption, *n* (%)	117 (17.3)	37 (16.4)	0.094	0.759
Underlying disease, *n* (%)				
Diabetes mellitus	154 (22.8)	42 (18.7)	1.705	0.192
Thyroid disorders	152 (22.5)	47 (20.9)	0.260	0.610
Cerebrovascular disease	101 (15.0)	32 (14.2)	0.074	0.786
Medical History, *n* (%)				
Head/face disorders	43 (6.4)	17 (7.6)	0.381	0.537
Psychiatric disorders	20 (3.0)	8 (3.6)	0.230	0.632
Antiparkinsonian drugs, *n* (%)	8 (1.2)	4 (1.8)	0.450	0.502

#### 3.4.1 Nomogram development

**Figure 1:**
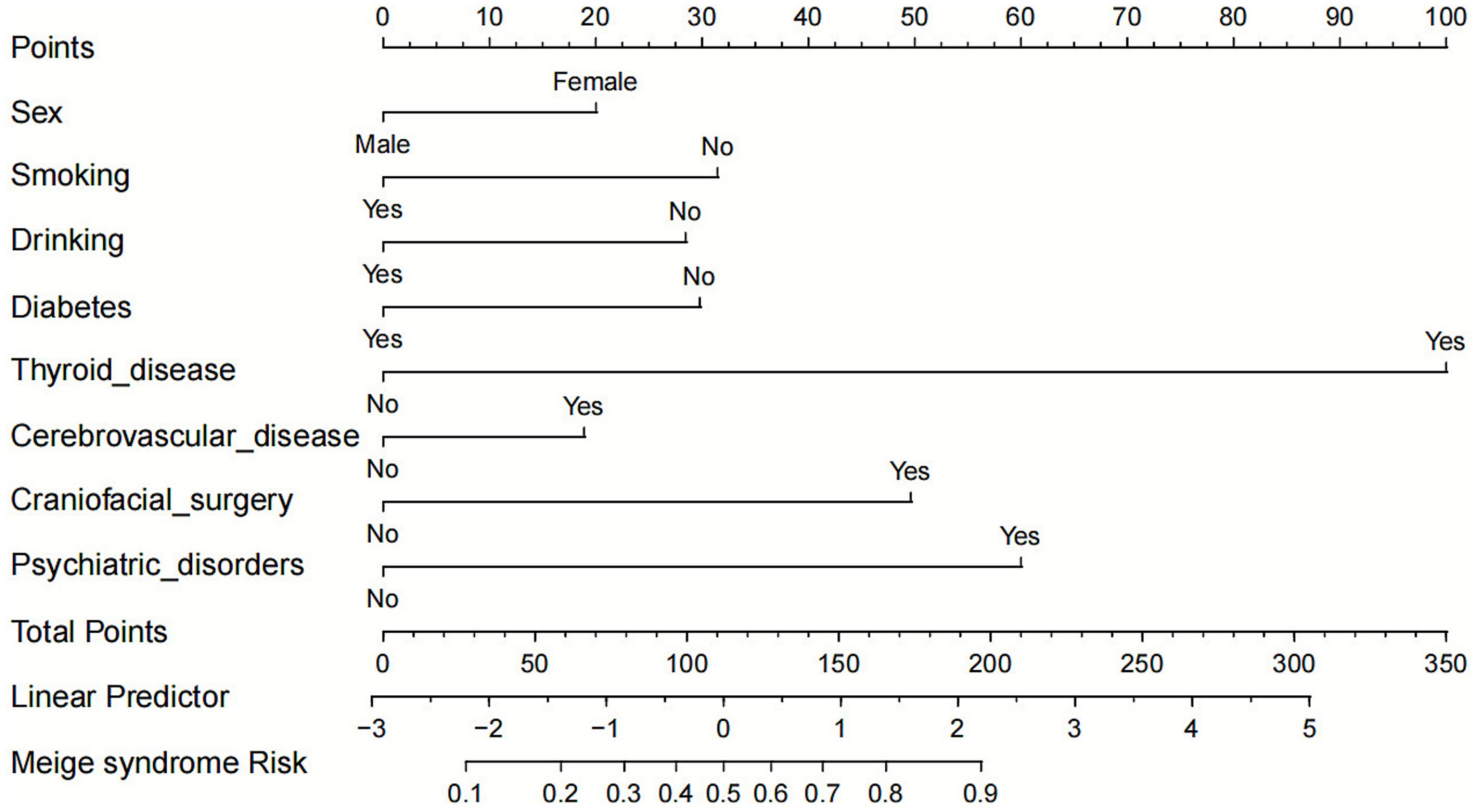
Nomogram model for predicting the risk of MS.

The nomogram incorporated eight independent predictors identified through multivariate analysis (sex, smoking history, alcohol consumption, diabetes history, thyroid disorders, cerebrovascular disease, head/face surgery history, and psychiatric history). This scoring system allocated points (total range: 0–350 points) based on variable contributions, with thyroid disorders (about 100 points), psychiatric history (about 80 points), and head/face surgery history (about 60 points) demonstrating the highest weight. Thyroid disorders are the strongest individual predictor in the model, suggesting a particularly significant association with MS risk relative to other included variables (Figure 1). To estimate an individual’s risk of MS using the nomogram, clinicians should first locate the patient’s value for each of the eight predictors on its corresponding variable axis (e.g., “Yes” for thyroid disorders) and draw a vertical line upward to the “Points” scale to read the assigned points. The points for all predictors are then summed to obtain a Total Points score, which is located on the bottom “Total Points” axis. Finally, a vertical line is drawn downward from the Total Points to the “Risk of MS” axis to read the corresponding predicted probability.

#### 3.4.2 Validation of the nomogram prediction model

**Figure 2:**
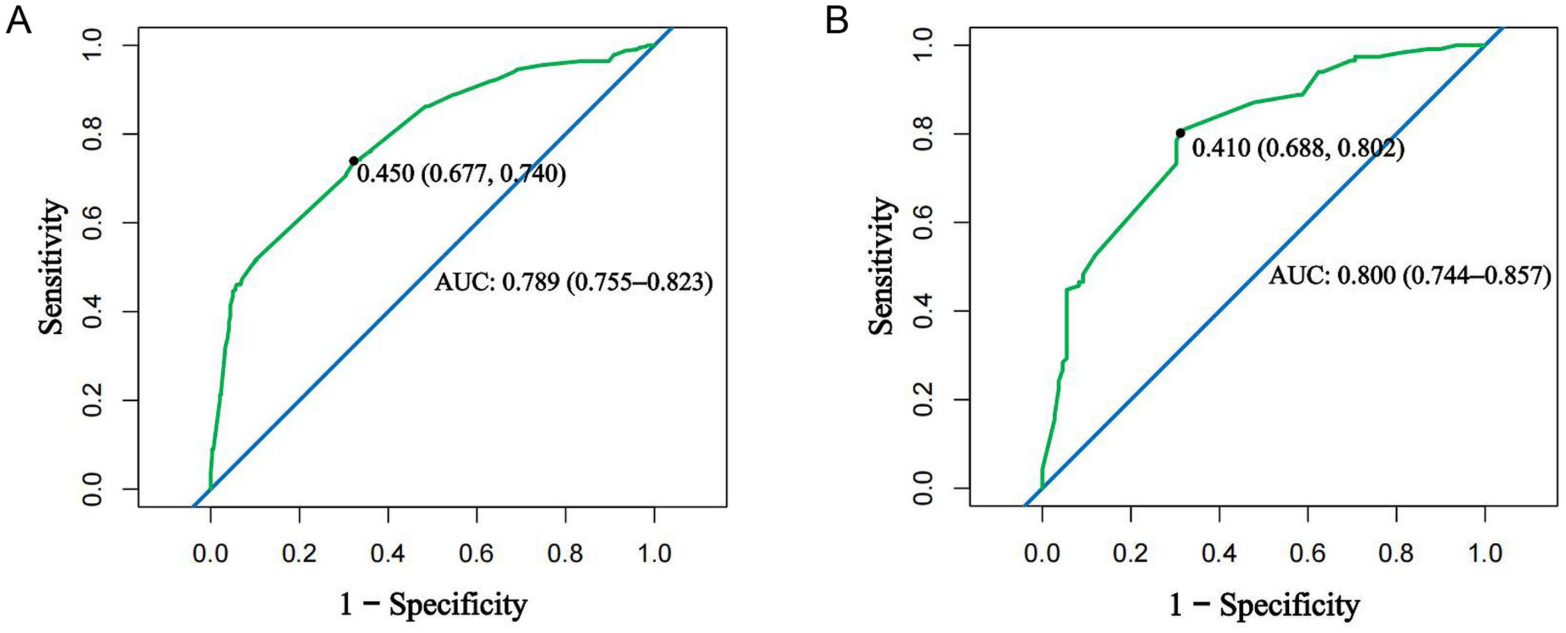
Receiver operating characteristic curves. **(A)** Training cohort; **(B)** validation cohort.

Model validation revealed excellent discrimination, with AUC values of 0.789 and 0.800 for training and validation cohorts, respectively. These values, both exceeding 0.75, indicate a robust ability to distinguish between patients with and without MS, confirming the model’s clinical utility for risk stratification. Using Youden-index-optimized cut-off values (0.450 in training and 0.410 in validation), the model achieved balanced diagnostic performance, with sensitivity and specificity of 75.2 and 66.1% in the training cohort, and 82.8 and 68.5% in the validation cohort. This balance ensures effective identification of true cases while maintaining a clinically acceptable specificity, supporting the model’s practical value as a screening or triage tool in clinical practice (Figure 2).

**Figure 3:**
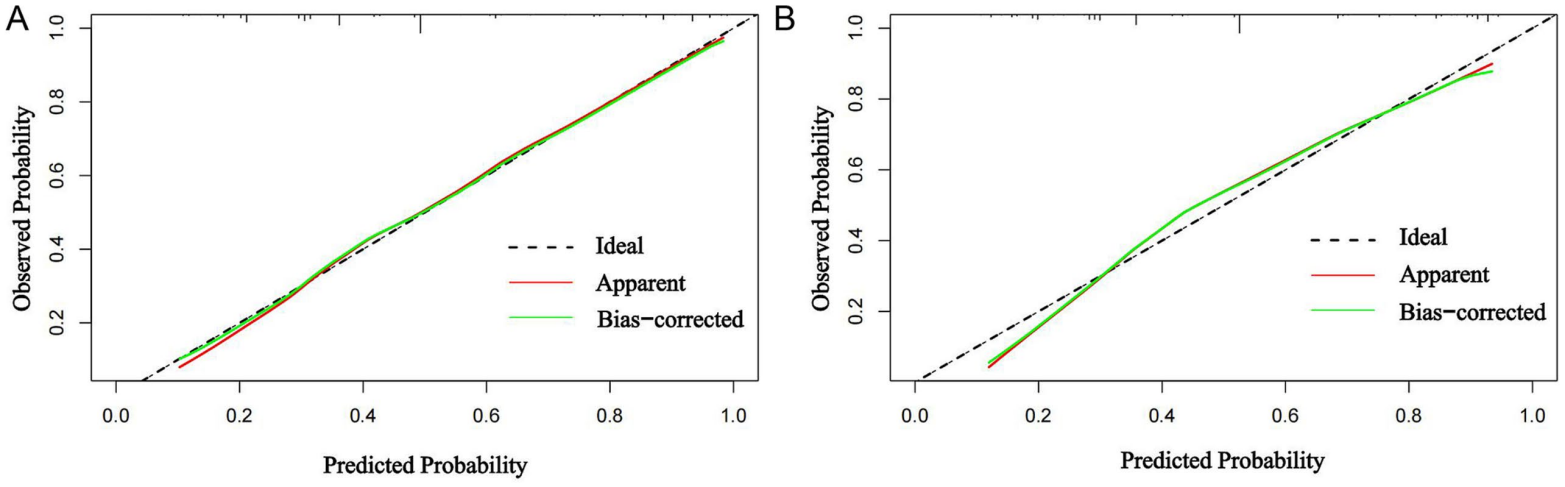
Calibration curves. **(A)** Training cohort; **(B)** validation cohort. Close agreement between the apparent/bias-corrected lines and the ideal curve demonstrates strong calibration accuracy, indicating reliable alignment of predicted and observed probabilities.

Calibration analysis (Figure 3) demonstrated a high degree of agreement between the model’s predicted probabilities and the observed outcomes, which reflects the goodness of fit of the nomogram. Specifically, the calibration curves for both the training and validation cohorts align closely with the ideal 45-degree line across the intermediate probability range (0.3–0.7). This close alignment indicates that the model’s predictions are accurate and reliable for the majority of risk strata. Minor deviations are observed in the extreme probability ranges (<0.3 and >0.8), where the predicted probabilities slightly diverge from the observed frequencies. These deviations are likely attributable to the relatively smaller sample size of patients within these very low- and very high-risk categories in our cohort, which can limit the precision of probability estimation in distributional tails.

**Figure 4:**
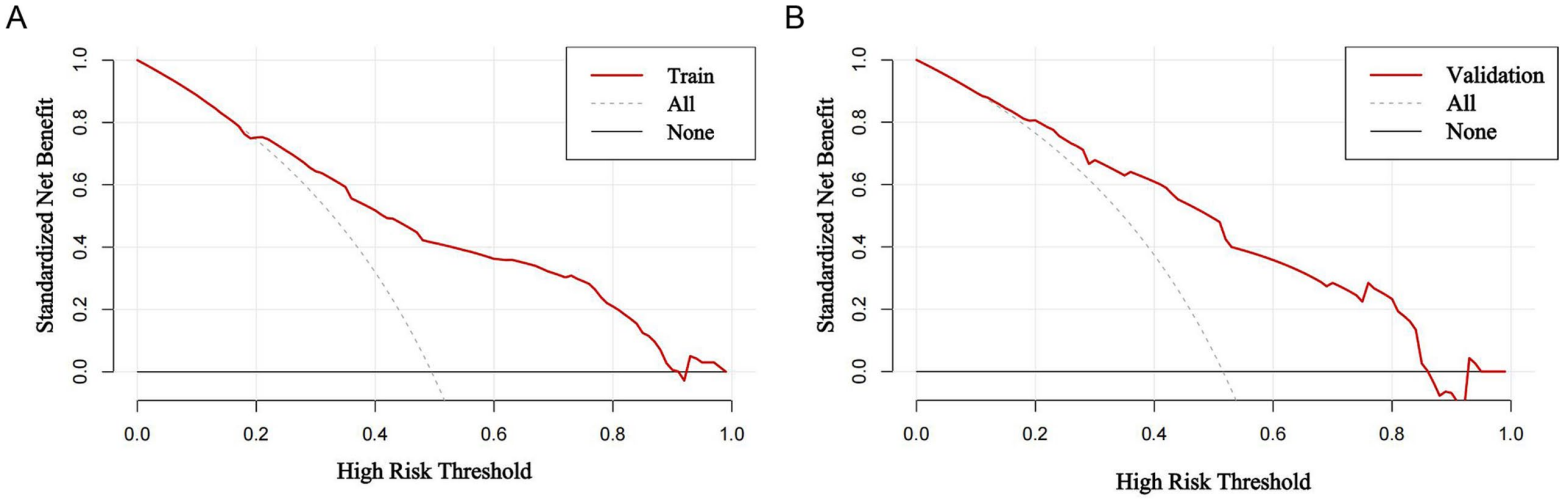
Decision curve analysis curves. **(A)** Training cohort; **(B)** validation group. All line: Represents the standardized net benefit when intervening in all patients suspected of MS; None line: Baseline scenario where no MS patients receive intervention. If the Train line was above both the all and none lines, indicating that the model performed better in the probability range. Clinical advantage persists even with false positives, as the benefits of early intervention outweigh the harms of unnecessary treatment in this probability range.

DCA (Figure 4) indicated that the prediction model provided a clear clinical net benefit across a wide threshold probability range of 0.2 to 0.8. The standardized net benefit curve was particularly outstanding within the 0.3–0.7 threshold interval, significantly outperforming the naive strategies of “treat all” or “treat none.” This suggests that when clinicians consider intervention warranted at a risk probability between 30 and 70%, applying this model for risk stratification maximizes net clinical benefit and supports more precise patient management. The high consistency between the training and validation cohort curves further confirms the robustness of this finding and the model’s strong generalizability.

## 4 Discussion

The clinical management of MS has long been confronted with the dual challenges of diagnostic delays and difficulties in determining optimal intervention timing, primarily due to inadequate understanding of the pathogenesis of this complex craniocervical dystonia. To address these pressing clinical issues, our study established the first clinical prediction nomogram for MS specifically tailored to the Chinese population. By systematically integrating both established risk factors and novelly identified protective factors (e.g., smoking, diabetes), the model offers a unique, multifactorial perspective on disease risk. The nomogram modeling approach innovatively transforms complex multifactorial interactions into a visual clinical decision-making tool, achieving a leap from basic research to clinical application. These findings not only provide a practical assessment tool for early identification of high-risk patients but, more importantly, open new avenues for exploring the etiological network of MS by establishing correlations between clinical indicators and neurobiological mechanisms.

Thyroid disorders were identified as the strongest independent risk factor, with an OR of 12.80. This value indicates that individuals with a history of thyroid disease have approximately 13 times higher odds of developing MS compared to those without such a history. Scorr et al. (10) identified a link between autoimmune thyroid diseases and various forms of focal dystonia, including craniocervical dystonia, and suggested that immune mechanisms may play a role. This pathophysiological link is thought to arise from a multisystem neural dysregulation, where immune-mediated processes and endocrine imbalances jointly disrupt the finely tuned neural circuits governing motor control. Thyroid toxicosis, characterized by excessive thyroxine levels due to various etiologies, disrupts balanced motor circuit modulation by altering dopaminergic and GABAergic neuronal activity within the basal ganglia-thalamocortical loop. Concurrently, it enhances acetylcholine receptor sensitivity at neuromuscular junctions, leading to hyperkinetic motor output (11–13). Conversely, hypothyroidism primarily induces inhibitory neurotransmission deficits (reduced GABA synthesis due to decreased GAD67 enzyme activity) (14) and abnormal myelination, impairing motor signal filtering. Both conditions collectively modulate mesolimbic dopaminergic system activity and trigger neuroinflammatory responses, ultimately affecting the motor-affective integration circuitry. This pathophysiological cascade manifests as the characteristic symptoms of blepharro-oro-mandibular segmental dystonia.

The robust association between psychiatric disorders (particularly anxiety disorders) and MS (OR = 6.89) is supported by a growing body of evidence and is likely driven by multilevel pathophysiological interactions. Ray et al. (15) also corroborated that 37.5 to 63.6% of patients with oromandibular dystonia and MS experience depression, and anxiety is similarly prevalent, which supported our findings. From a neural circuitry perspective, anxiety-related amygdala hyperactivation directly enhances inhibitory pallidal projections, disrupting the basal ganglia’s motor signal filtering capacity, while concurrently activating the hypothalamic-locus coeruleus-noradrenergic system (16). This dual dysregulation increases spinal motor neuron excitability, facilitating the release of abnormal movements (e.g., blepharospasm). At the neurotransmitter level, both anxiety patients and MS patients exhibit reduced cortical GABA concentrations, further compromising inhibitory control over motor cortices (17). Notably, the resulting “psychomotor positive feedback loop” plays a pivotal role: patients develop body dysmorphic disorder and social anxiety due to facial dystonia, and this psychological stress exacerbates motor symptoms via heightened limbic system activity, creating a self-reinforcing vicious cycle (15). These findings not only clarify anxiety’s role as an independent risk factor but also provide a mechanistic rationale for combined neuromodulation and psychological interventions in clinical management.

A history of craniofacial surgery emerged as another independent risk factor for MS (OR = 3.47). The underlying mechanism may involve direct intraoperative damage to facial and trigeminal nerve branches during procedures such as parotidectomy or orthognathic surgery (18). Such injuries can induce both peripheral nerve hyperexcitability and compensatory reorganization of central sensorimotor cortices, manifesting as contraction of facial representation areas in the somatosensory cortex and enhanced functional connectivity in motor cortices (19). These neural insults concurrently impair sensory gating mechanisms, reducing thalamic filtering capacity for sensory inputs and resulting in abnormal amplification of ordinary facial tactile stimuli. Crucially, postoperative psychological stress may further amplify motor circuit excitability through activation of the amygdala-locus coeruleus pathway, establishing a self-reinforcing “neural injury-sensory dysfunction-psychological stress” cycle. This triad explains the significantly elevated risk of dystonia following craniofacial surgeries and identifies multiple potential intervention targets for clinical prevention and treatment (20).

Female sex (OR = 1.87) and cerebrovascular disease history (OR = 2.00) emerge as risk factors for MS through a complex “biphasic modulation” mechanism. Estrogen exerts concentration-dependent dual effects on the motor system: at physiological concentrations, it provides neuroprotection by maintaining basal ganglia GABAergic neuron activity and cerebrovascular endothelial function, while abnormally elevated or fluctuating levels excessively stimulate dopamine D2 receptors, leading to motor inhibition dysfunction (21). This mechanism explains both the markedly higher incidence in women and the critical perimenopausal transition period, where estrogen fluctuations simultaneously eliminate neuroprotective effects and prevent compensatory adaptation (22). Concurrently, age-related cerebrovascular pathologies (e.g., lacunar infarcts) further damage basal ganglia-thalamic circuits. The synergistic interplay of “neuroprotection loss and vascular injury” precipitates dystonia, resulting in dramatically elevated risk in postmenopausal women with cerebrovascular disease.

Although thyroid disorders, psychiatric history, and craniofacial surgery demonstrate strong associations with MS, their mechanistic underpinnings require further validation. Conversely, the identification of protective factors like smoking offers novel preventive insights. Current evidence reveals significant inverse correlations between smoking (OR = 0.411), alcohol consumption (OR = 0.396), diabetes (OR = 0.534), and MS risk, mediated through complex neurobiological regulatory networks. Nicotine, a selective agonist of nicotinic acetylcholine receptors, exerts dual modulation of basal ganglia motor circuits: By activating striatal cholinergic interneurons, it suppresses GABAergic neuron-mediated inhibition of the globus pallidus externa (GPe) via the indirect pathway, thereby enhancing GPe-mediated suppression of the subthalamic nucleus (23–25); Concurrently, it reduces excessive inhibitory output from the globus pallidus interna to the thalamus, ultimately rebalancing motor output – a mechanism potentially alleviating MS-related hyperkinesia. Additionally, nicotine stimulates ventral tegmental area dopaminergic neurons, augmenting mesolimbic dopamine pathway activity (26–28). This not only improves affective states but may also mitigate MS symptom severity through motor-affective circuit modulation. Ethanol’s protective effects primarily stem from positive allosteric modulation of GABA-A receptors, enhancing central inhibitory neurotransmission to counteract motor circuit hyperexcitability (29). Low-dose ethanol further attenuates hypothalamic–pituitary–adrenal axis hyperactivity, reducing catecholamine-driven motor circuit excitation – a mechanism particularly relevant for stress-induced symptom exacerbation (30, 31). Notably, diabetes confers protection through unique peripheral sensory mechanisms. Given that trigeminal sensitization-mediated blink reflex hyperexcitability constitutes a core pathological feature of MS, chronic hyperglycemia-induced metabolic derangements (e.g., intra-neuronal osmotic imbalance, mitochondrial oxidative phosphorylation dysfunction, and free radical accumulation) impair ophthalmic branch fiber structure/function (32). These changes reduce corneal nerve sensitivity, dampening blink reflex afferent signals and disrupting the “sensory sensitization-motor hyperactivity” cascade – a phenomenon indirectly corroborated by the clinical efficacy of trigeminal nerve blocks in refractory blepharospasm (33). It should be emphasized that these ostensibly “protective” effects represent compensatory adaptations to pathological states. The neurological benefits of smoking/alcohol are counterbalanced by systemic health risks, while diabetes-mediated neural inhibition cannot offset its vascular complications. The true significance lies in identifying nAChRs, GABAergic systems, and sensory afferent pathways as potential therapeutic targets – rather than endorsing these lifestyle factors themselves (34).

This study developed and validated a novel nomogram incorporating eight independent predictors (gender, smoking, alcohol use, diabetes, thyroid disorders, cerebrovascular disease, craniofacial surgery, and psychiatric history) for MS risk stratification. The model demonstrated robust predictive performance, with AUCs of 0.789 in the training set and 0.800 in the validation set, indicating excellent discriminative ability. Calibration analysis revealed strong agreement between predicted and observed probabilities, particularly in the clinically relevant 0.3–0.7 risk range. DCA confirmed the model’s clinical utility, showing significant net benefit across threshold probabilities of 0.2–0.8. By converting these predictors into a visual, points-based scoring system (total score range: 0–350 points), the nomogram translates complex risk interactions into an individualized probability estimate (0.1–0.9). This provides clinicians with an intuitive tool for early risk stratification in patients presenting with suggestive symptoms. In practice, a patient’s total points—heavily influenced by factors such as thyroid disorders (100 points), psychiatric history (80 points), and craniofacial surgery (60 points)—can be quickly calculated during routine evaluation. The corresponding risk probability can then guide decisions regarding further diagnostic work-up, specialist referral, or patient counseling, thereby facilitating targeted and timely interventions.

This study has several limitations that warrant consideration. First, the single-center retrospective design resulted in notable geographical imbalance. Although we enrolled 1,041 MS patients from 29 provincial-level regions across China, nearly half of the cohort originated from the hospital’s home province (with Henan Province accounting for 42.2% of cases while no other single region exceeded 4.7%). This distribution may reflect regional patient referral patterns to our tertiary care center, the overrepresentation of Henan cases (*n* = 439) could introduce selection bias, particularly in evaluating region-specific factors such as environmental exposures or healthcare accessibility. Consequently, the model requires external validation in diverse, multicenter, and ideally prospective cohorts to confirm its generalizability across different populations and settings. Secondly, our validation cohort (*n* = 225) was relatively small and lacked completely independent external validation data, which may compromise the generalizability of our prediction model across diverse geographical populations. Future multi-center studies with larger, prospective cohorts are essential to perform rigorous external validation and to evaluate the model’s stability in different healthcare settings. Thirdly, regarding protective factors, the observed inverse associations with smoking and alcohol consumption might be influenced by survival bias, as patients with comorbid conditions like chronic obstructive pulmonary disease may have been lost to follow-up. Further prospective research is needed to clarify the nature of these associations and to determine whether they represent true protective effects or artifacts of study design. Fourth, while thyroid disorder history emerged as the strongest predictor, our inability to differentiate subtypes (e.g., Hashimoto’s thyroiditis vs. Graves’ disease) or analyze antibody levels precludes definitive conclusions about the contribution of autoimmune mechanisms. Finally, the retrospective case–control design of this study carries a potential risk of reverse causality. For instance, the observed inverse associations between smoking, alcohol consumption, and MS could theoretically arise from behavioral changes prompted by early, undiagnosed symptoms of MS, rather than representing a causal protective effect of these exposures. However, considering the typically insidious onset of MS and the fact that smoking and drinking are often long-term habits, this alternative explanation may have limited plausibility. Nevertheless, the precise causal direction of all observed associations, particularly those involving strong risk factors such as thyroid disorders and psychiatric history, requires confirmation through future prospective cohort studies. Future studies incorporating detailed thyroid function tests, antibody panels, and mechanistic investigations are warranted to elucidate the potential role of autoimmune dysregulation in the pathogenesis of MS.

## 5 Conclusion

This study presents a novel clinical prediction nomogram for MS, integrating both risk and protective factors into a practical, visual tool. The model demonstrates strong predictive performance, clinical utility, and calibration, offering a validated approach for early risk stratification and individualized intervention planning. Our findings not only advance the understanding of MS etiology by confirming key factors such as thyroid disorders, psychiatric history, and craniofacial surgery, but also provide clinicians with a quantitative means to translate complex risk profiles into actionable probabilities. To ensure broader clinical applicability, future multicenter prospective studies are needed to externally validate and further refine this predictive tool.

## Data Availability

The datasets presented in this article are not readily available because the datasets generated during the current study are not publicly available due to patient privacy and ethical concerns. Requests to access the datasets should be directed to XL, 1776839026@qq.com.
